# Lymph node ratio is a more robust predictor of overall survival than N stage in stage III colorectal adenocarcinoma

**DOI:** 10.1186/s13000-024-01449-6

**Published:** 2024-02-28

**Authors:** Amir F. Beirat, Justin Z. Amarin, Haya H. Suradi, Yasmeen Z. Qwaider, Adel Muhanna, Bayan Maraqa, Abdallah Al-Ani, Maysa Al-Hussaini

**Affiliations:** 1 Office of Scientific Affairs and Research, King Hussein Cancer Center, Amman, 11941 Jordan; 2 Istishari Hospital, Amman, 11184 Jordan; 3 Department of General and Gastrointestinal Surgery, Massachusetts General Hospital, Harvard Medical School, Boston, MA 02114 USA; 4 Department of Internal Medicine, University of Missouri–Kansas City, Kansas City, MO 64110 USA; 5 Department of Pathology and Laboratory Medicine, King Hussein Cancer Center, Amman, 11941 Jordan

**Keywords:** Adenocarcinoma, Colorectal neoplasms, Lymph node ratio, Prognosis, Survival analysis

## Abstract

**Background:**

Lymph node ratio (LNR) may offer superior prognostic stratification in colorectal adenocarcinoma compared with N stage. However, candidate cutoff ratios require validation. We aimed to study the prognostic significance of LNR and its optimal cutoff ratio.

**Methods:**

We reviewed the pathology records of all patients with stage III colorectal adenocarcinoma who were managed at the King Hussein Cancer Center between January 2014 and December 2019. We then studied the clinical characteristics of the patients, correlates of lymph node count, prognostic significance of positive lymph nodes, and value of sampling additional lymph nodes.

**Results:**

Among 226 included patients, 94.2% had ≥ 12 lymph nodes sampled, while 5.8% had < 12 sampled lymph nodes. The median number of lymph nodes sampled varied according to tumor site, neoadjuvant therapy, and the grossing pathologist’s level of training. According to the TNM system, 142 cases were N1 (62.8%) and 84 were N2 (37.2%). Survival distributions differed according to LNR at 10% (*p* = 0.022), and 16% (*p* < 0.001), but not the N stage (*p* = 0.065). Adjusted Cox-regression analyses demonstrated that both N stage and LNR at 10% and 16% predicted overall survival (*p* = 0.044, *p* = 0.010, and *p* = 0.001, respectively).

**Conclusions:**

LNR is a robust predictor of overall survival in patients with stage III colorectal adenocarcinoma. At a cutoff ratio of 0.10 and 0.16, LNR offers better prognostic stratification in comparison with N stage and is less susceptible to variation introduced by the number of lymph nodes sampled, which is influenced both by clinical variables and grossing technique.

## Background

Colorectal cancer is the third most common cancer and the second leading cause of cancer death worldwide [1]. Surgery is the mainstay of treatment and involves the resection of affected bowel segments and the draining of lymph nodes. Surgical specimens are crucial for staging and prognostication, and the results of their analysis help guide postoperative management [2–4]. According to a consensus statement by the College of American Pathologists, a minimum of 12 lymph nodes should be sectioned because 12–15 negative lymph nodes predict no regional lymph node involvement [5].

The number of positive lymph nodes is an important determinant of the stage of colorectal cancer according to the TNM staging system of the American Joint Committee on Cancer and the Union for International Cancer Control (AJCC/UICC). Other nodal characteristics, such as the total number of lymph nodes sampled, nodal distribution, and lymph node size, are also prognostically significant [6–10]. In addition, many studies have shown that lymph node ratio (LNR), which is the number of positive lymph nodes divided by the total number of lymph nodes sampled, is also prognostically significant [7, 11, 12]. Ceelen et al. performed a systematic review of 16 studies and found that LNR is an independent prognostic factor in stage III colorectal cancer and offers superior prognostic stratification compared with the N stage of the TNM staging system [13]. However, an “optimal” cutoff ratio for the interpretation of LNR has not been identified as its value varies widely from study to study. In their systematic review, Ceelen et al. found that the median LNR was approximately 0.10, and they recommend the use of this value as the cutoff ratio [13]. We therefore aimed to study the prognostic significance of LNR and determine its most optimal cut-off value.

## Methods

We performed a retrospective chart review of all patients with colorectal adenocarcinoma who were managed at the King Hussein Cancer Center (KHCC) between January 2014 and December 2019. KHCC is a Joint Commission International (JCI)-accredited cancer center in Amman, Jordan that serves roughly 60% of all patients with cancer in Jordan [14]. We included patients with stage III disease and retrieved the following data from the center’s Cancer Registry as well as pathology reports: age, sex, tumor site, resection length, neoadjuvant therapy, TNM stage, grade, histologic subtype, the grossing pathologist’s sex (i.e., male or female), and the grossing pathologist’s level of training. We also retrieved date of surgery, date last seen, and overall survival (OS) status from our institution’s electronic medical records. To address data missingness or outdatedness, we requested and received additional survival data from the National Civil Status and Passports Department, a government agency that curates current survival data. The Institutional Review Board (IRB) of KHCC, which complies with the Declaration of Helsinki and the Good Clinical Practice guidelines, reviewed and approved the study protocol (20KHCC10). The IRB waived the requirement for informed consent because the study involves existing data and no interaction with participants.

### Statistical methods

We used R (version 4.0.2) to perform data analysis. First, we described the clinical characteristics of the cohort. We then studied the correlation between the number of lymph nodes sampled and continuous variables using Spearman’s *ρ*. We also studied differences in the number of lymph nodes sampled between the groups of categorical variables using the Mann–Whitney *U* test. The prognostic capacity of LNR was initially explored using Receiver Operating Characteristic (ROC) analysis to determine its best possible cut-off value. Next, we used the Kaplan–Meier method to plot two sets of OS curves by N stage (N1 versus N2), LNR (< 0.10 versus ≥ 0.10), and LNR (< 0.16 versus ≥ 0.16) and estimated the 1- and 5-year OS rates. Per the AJCC/UICC’s TNM classification system, the N1 stage refers to metastasis in 1 to 3 regional lymph nodes or the presence of tumor deposits. Conversely, N2 staging refers to metastasis in 4 or more regional lymph nodes [15]. We defined OS as the time from the date of surgery to the date last seen or the date of death from any cause. We also compared the OS curves in each set using the log-rank test. We then fit Cox regression models to predict OS as a function of either N stage or LNR along with a set of six other predictors—namely: age, sex, tumor site, neoadjuvant therapy, grade, and histologic subtype. Finally, we examined cases in which additional lymph nodes were sampled and described the effect of revision on the N stage and LNR. For all hypothesis tests, we interpreted values of *p* ≤ 0.05 to indicate statistical significance.

## Results

Our service processed specimens from 592 patients with colorectal adenocarcinoma between January 2014 and December 2019. The disease was stage 0 in 20 cases (3.4%; seven cases in situ [1.2%]), stage I in 72 (12.2%), stage II in 216 (36.5%), stage III in 226 (38.2%), and stage IV in 58 (9.8%). We limited subsequent analyses to the 226 patients with stage III disease.

### Summary statistics

The mean age of the 226 patients with stage III disease was 58.2 ± 13.8 years (range, 21–86 years). Men (*n* = 123, 54.4%) outnumbered women (*n* = 103, 45.6%) with a male-to-female ratio of 1.2-to-1. The primary tumor site was the cecum and ascending colon in 33 cases (14.6%), hepatic flexure in six (2.7%), transverse colon in 19 (8.4%), splenic flexure in three (1.3%), descending colon in four (1.8%), sigmoid colon in 46 (20.4%), and rectum in 115 (50.9%). The median resection length was 23 cm (range, 5–140 cm). Seventy patients (31.0%) received neoadjuvant therapy and 156 (69.0%) did not. Tumors were well-differentiated in two cases (0.9%), moderately differentiated in 183 cases (81.0%), and poorly differentiated in 40 cases (17.7%). The grade of one regressed tumor (0.4%) could not be assessed. Of all tumors, 180 (79.6%) were classic adenocarcinomas and 46 (20.4%) were mucinous adenocarcinomas.

### Correlates of the number of lymph nodes sampled

The specimens yielded a total of 5,649 lymph nodes (median, 21.5; range, 3–147). The number of lymph nodes sampled was < 12 in 13 cases (5.8%) and ≥ 12 in 213 (94.2%). Higher lymph node counts were correlated with younger patient age (*ρ* = −0.16; *p* = 0.014) and lengthier resections (*ρ* = 0.18; *p* = 0.007). In addition, the median number of lymph nodes sampled differed according to tumor site, neoadjuvant therapy, and the grossing pathologist’s level of training, but not patient sex, tumor grade, histologic subtype, or the grossing pathologist’s sex (Table 1). Briefly, the median number of lymph nodes sampled was higher if the tumor site was the colon, the patient had not received neoadjuvant therapy, or the specimen was handled by a fellow or specialist.

**Table 1:** Median numbers of lymph nodes sampled according to the characteristics of the patient, tumor, and grossing pathologist (*N* = 226 patients with stage III colorectal adenocarcinoma)

Characteristics	Median (MAD)	*p* value
**Sex**		0.16
* Female*	22.0 (8.9)	
* Male*	21.0 (7.4)	
**Tumor site**		**< 0.001**
* Rectum*	19.0 (5.9)	
* Colon*	25.0 (8.9)	
**Neoadjuvant therapy**		**< 0.001**
* No neoadjuvant therapy*	24.0 (8.9)	
* Chemotherapy and/or radiotherapy*	18.0 (4.5)	
**Grade**		0.52
* Well-differentiated or moderately differentiated*	22.0 (8.9)	
* Poorly differentiated*	21.5 (7.4)	
**Histologic subtype**		0.73
* Classic*	22.0 (8.9)	
* Mucinous*	21.0 (8.9)	
**Grossing pathologist’s sex**		0.084
* Female*	23.0 (8.9)	
* Male*	20.0 (7.4)	
**Grossing pathologist’s level of training**		**0.010**
* Resident*	21 (7.4)	
* Fellow or specialist*	22 (8.9)	

Abbreviations: MAD, median absolute deviation

### Prognostic significance of positive lymph nodes

According to the TNM system, 142 cases were N1 (62.8%) and 84 were N2 (37.2%). Of the N1 cases, 61 (43.0%) were N1a, 68 (47.9%) were N1b, and 13 (9.2%) were N1c. Of the N2 cases, 41 (48.8%) were N2a and 43 (51.2%) were N2b. The total number of positive lymph nodes was 970 (out of 5,649) for a total LNR of 0.17. The median number of positive lymph nodes was 2 , and the median LNR was 0.11 . The LNR was < 0.10 in 98 cases (43.4%) and ≥ 0.10 in 128 (56.6%). Survival data were available for 211 patients (93.4%) who were nationals of Jordan; nationality accounted for all data missingness because of the method we used to retrieve survival data. The 211 patients were followed for 6,804 person-months (median, 28 months). During the follow-up period, 36 patients (17.1%) died.

**Fig. 1:**
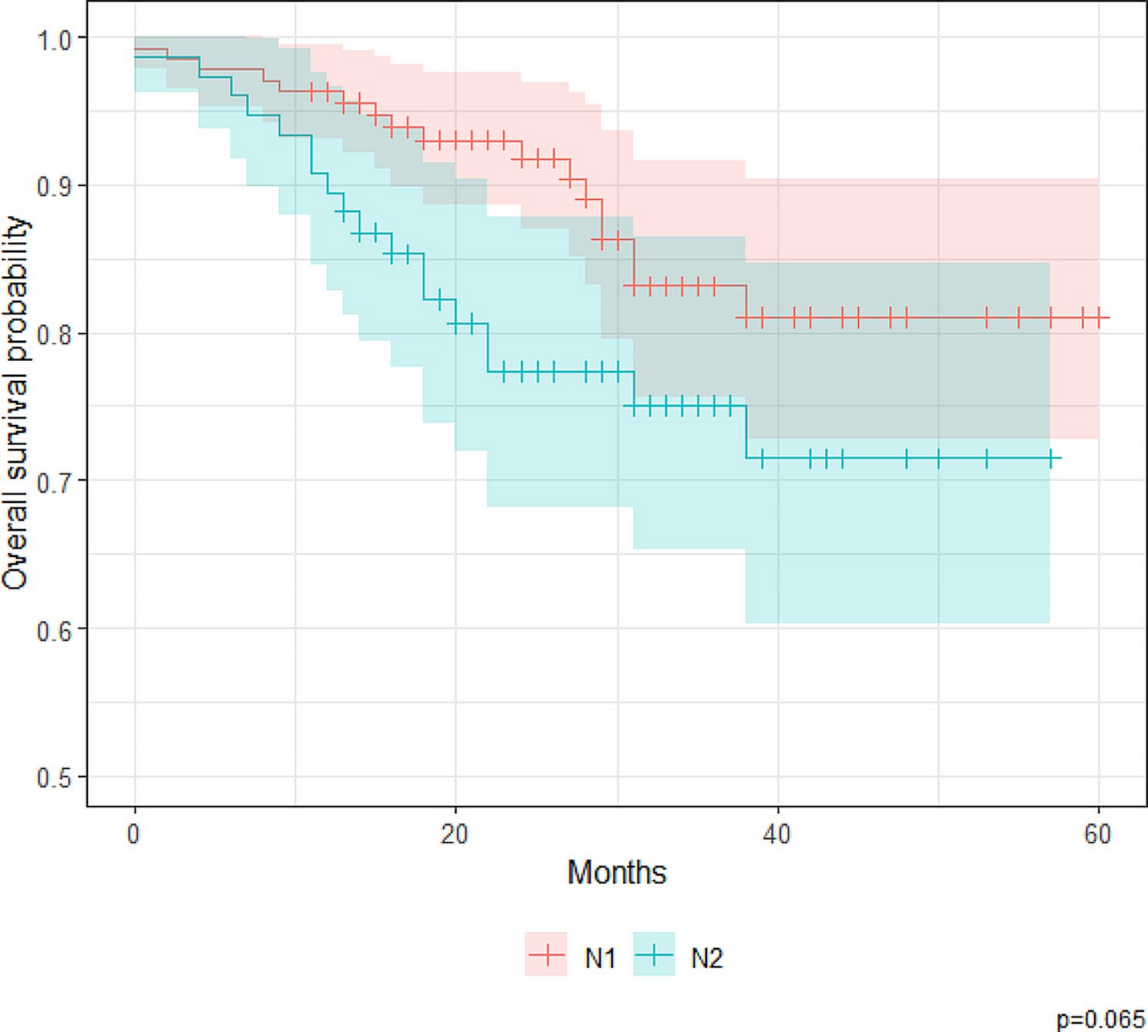
Kaplan–Meier estimates of overall survival, stratified by N stage (*N* = 211 patients with stage III colorectal adenocarcinoma)

**Fig. 2:**
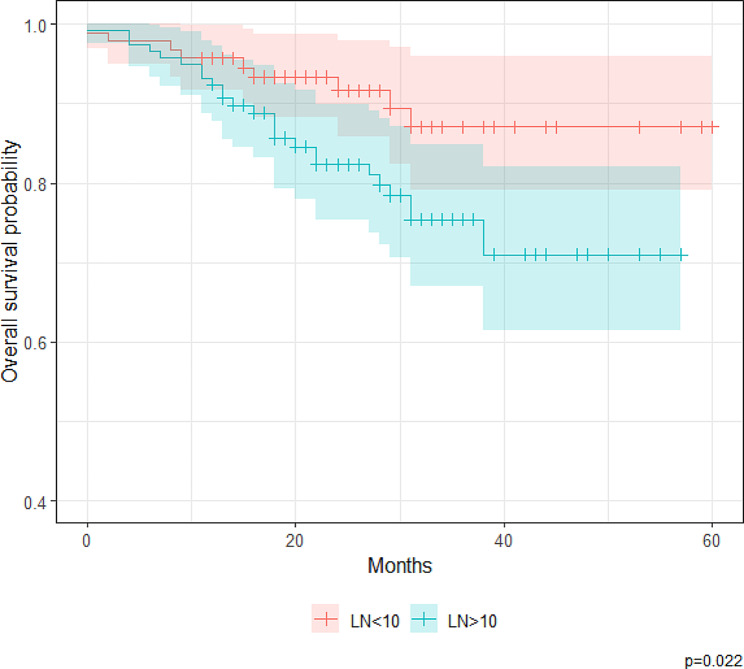
Kaplan–Meier estimates of overall survival, stratified by the lymph node ratio at a cutoff ratio of 0.10 (*N* = 211 patients with stage III colorectal adenocarcinoma)

Figures 1 and 2 show the OS curves of the patients according to N stage (N1 versus N2) and LNR (< 0.10 versus ≥ 0.10), respectively. Survival distributions significantly differed according to LNR (*p* = 0.022) but not N stage (*p* = 0.065). The 1- and 5-year OS rates for patients with an LNR < 0.10 were 95.7% (95% CI, 91.7–99.9%) and 87.1% (95% CI, 79.0–95.9%), respectively. In comparison, the 1- and 5-year OS rates for patients with an LNR ≥ 0.10 were 92.4% (95% CI, 87.7–97.3%) and 71.0% (95% CI, 61.4–82.0%), respectively.

### ROC curve analysis and LNR cut-off determination

**Fig. 3:**
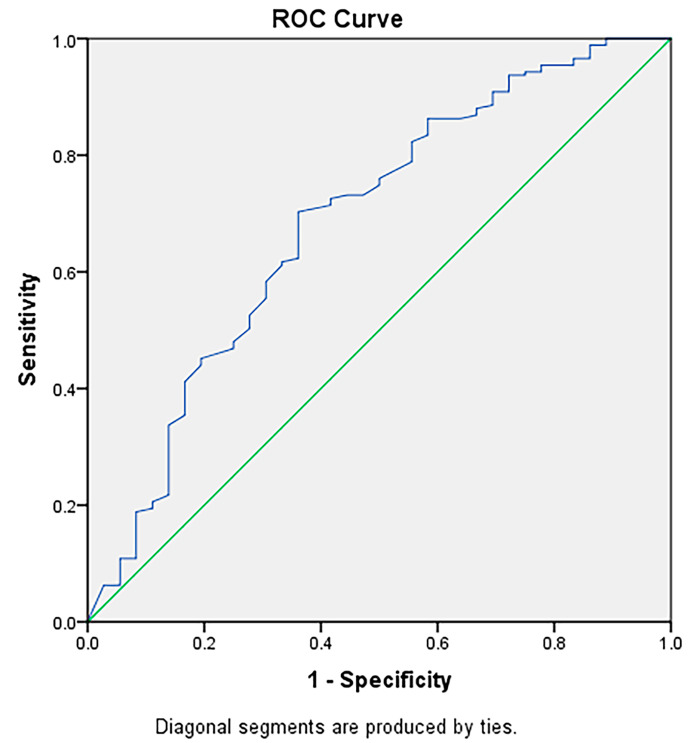
ROC analysis of LNR

**Fig. 4:**
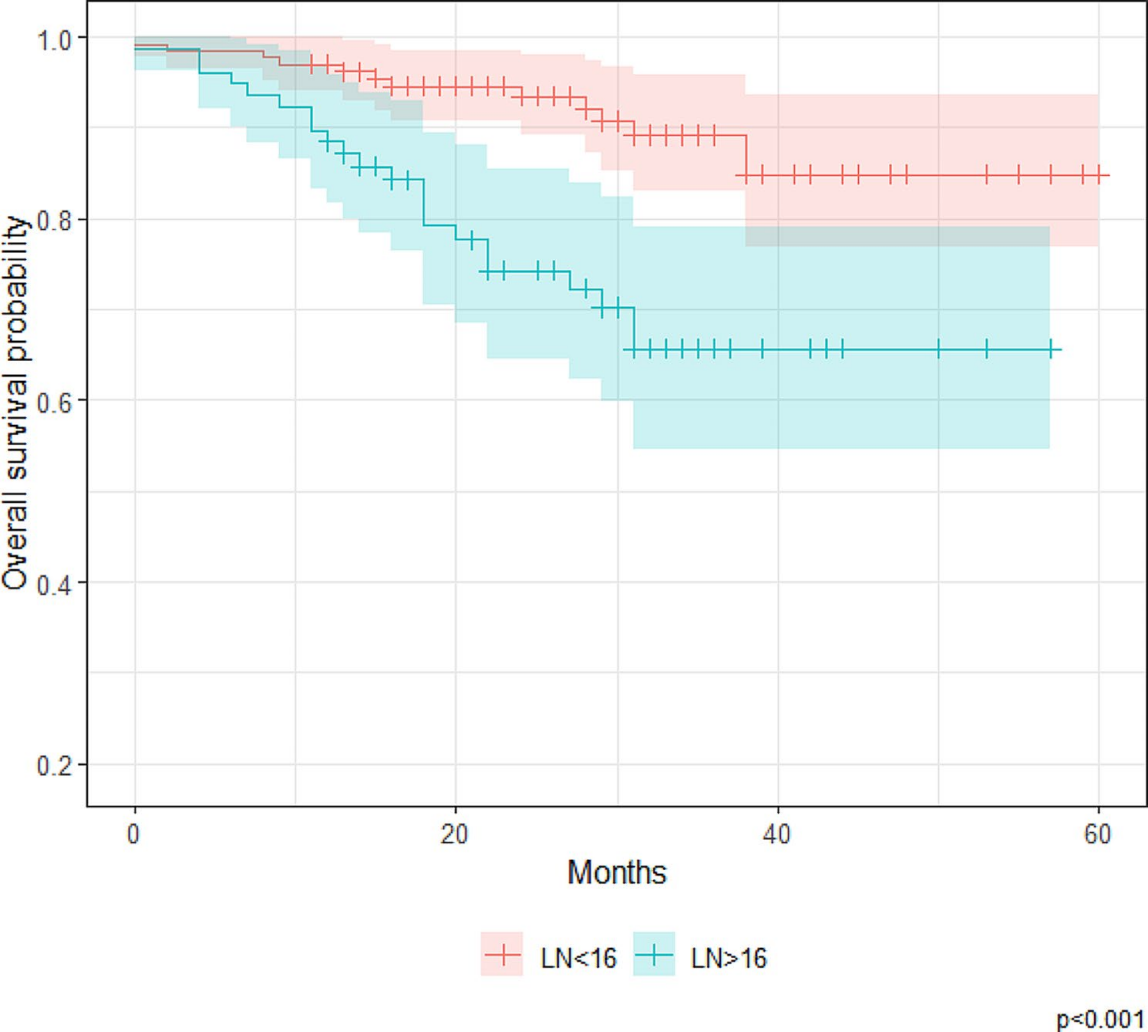
Kaplan–Meier estimates of overall survival, stratified by the lymph node ratio at a cutoff ratio of 0.16 (*N* = 211 patients with stage III colorectal adenocarcinoma)

When undergoing ROC analysis, an LNR of 16% (sensitivity: 64.0%; specificity: 70.3%; negative predictive value: 90.4%; positive predictive value: 30.7%) demonstrated higher accuracy than an LNR of 10% (sensitivity: 72.2%; specificity; 50.3%; negative predictive value: 89.8%; positive predictive value: 23.0%). Figure 3 shows the area under the curve for the ROC curve (AUC:0.685 [95% CI: 0.584–0.787]). Survival distribution remained significantly different per the optimized LNR cut-off of 16% (*p* < 0.01) (Fig. 4). The 1- and 5-year OS rates for patients with an LNR < 0.16 were 96.9% (95% CI, 94.1 – 99.9%) and 84.9% (95% CI, 76.8 – 93.7%), respectively. In comparison, the 1- and 5-year OS rates for patients with an LNR ≥ 0.16 were 88.5% (95% CI, 81.6 – 95.8%) and 65.7% (95% CI, 54.5 – 79.1%), respectively.

### Multivariate analysis

Cox regression demonstrates that the N stage (HR: 2.0; 95% CI, 1.0–4.0), LNR at 10% stage (HR: 2.8; 95% CI, 1.3–6.0), and LNR at 16% stage (HR: 3.7; 95% CI, 1.9–7.4) were able to significantly predict OS (*p* = 0.044, *p* = 0.010, and *p* = 0.001, respectively) (Table 2). Other predictors of OS included tumor site, neoadjuvant therapy, and tumor grade.

**Table 2:** Cox regression models of overall survival as a function of either N stage or lymph node ratio (LNR) and other established predictors of worse prognosis (*N* = 211 patients with stage III colorectal adenocarcinoma)

Predictors	Model with N stage		Model with LNR (10%)		Model with LNR (16%)	
	HR (95% CI)	*p* value	HR (95% CI)	*p* value	HR (95% CI)	*p* value
**N stage**		**0.044**		NA		NA
N1	Reference		NA		NA	
N2	2.02 (1.02–4.00)		NA		NA	
**LNR (10%)**		NA		**0.010**		NA
< 10	NA		Reference		NA	
≥ 10	NA		2.76 (1.28–5.96)		NA	
**LNR (16%)**		NA		NA		**0.001**
< 16	NA		NA		Reference	
≥ 16	NA		NA		3.70 (1.86–7.36)	
**Age**	1.03 (1.00–1.05)	0.066	1.02 (1.00–1.05)	0.074	1.03 (1.00–1.05)	0.058
**Sex**		0.25		0.21		0.27
Female	Reference		Reference		Reference	
Male	1.52 (0.75–3.09)		1.57 (0.77–3.18)		1.49 (0.73–3.01)	
**Tumor site**		**0.035**		**0.014**		**0.026**
Rectum	Reference		Reference		Reference	
Colon	3.17 (1.08–9.30)		4.1 (1.3–12.9)		3.66 (1.17–11.4)	
**Neoadjuvant therapy**		**0.012**		**0.010**		**0.011**
No neoadjuvant therapy	Reference		Reference		Reference	
Chemotherapy and/or radiotherapy	4.1 (1.4–12.2)		4.6 (1.4–14.9)		4.49 (1.40–14.3)	
**Grade**		**0.026**		**0.016**		**0.028**
Well-differentiated or moderately differentiated	Reference		Reference		Reference	
Poorly differentiated	2.77 (1.36–6.76)		3.02 (1.23–7.40)		2.67 (1.11–6.41)	
**Histologic subtype**		0.089		0.091		0.090
Classic	Reference		Reference		Reference	
Mucinous	0.43 (0.16–1.14)		0.44 (0.17–1.14)		0.44 (0.17–1.14)	

Abbreviations: HR, hazard ratio; CI, confidence interval

### Sampling of additional lymph nodes

Our service processed additional specimens in nine cases (4.0%). The specimens yielded a total of 48 lymph nodes (median, 5; range, 1–11). The total number of positive lymph nodes was 7 for a total LNR of 0.15. The N stage of one case (11.1%) was revised from N2a to N2b. The classification of the remaining cases was not revised. The initial total LNR of the nine cases was 0.18 and the final total LNR was 0.17. None of the cases crossed the 0.10 ratio threshold following revision.

## Discussion

We explored the clinicopathologic characteristics of 226 patients with stage III colorectal adenocarcinoma and studied the correlates of the number of lymph nodes sampled, the prognostic significance of positive lymph nodes, and the value of sampling additional lymph nodes. We found that the number of lymph nodes sampled was higher for patients who were younger, had lengthier resections, had a tumor of the colon, had not received neoadjuvant therapy, and whose specimens were handled by a fellow or a specialist. We also found that, compared with N stage, LNR was a more robust predictor of OS. Finally, we examined nine cases in which additional lymph nodes were sampled and found that the N stage of one case was revised but the LNR category of none of the cases was revised.

Several factors determine the number of lymph nodes sampled. Bamboat et al. showed that first-year pathology residents sampled a higher number of lymph nodes than residents with more years of training [16]. Kuijpers et al. also showed that pathology assistants sampled a higher number of lymph nodes than pathologists, and they attributed this finding to differences in time allocation for sampling and numbers of distractions between pathologists and pathology assistants [17]. However, we found that fellows and specialists sampled a higher number of lymph nodes than residents. In support of the aforementioned, previous studies have shown that additional training and a standard protocol improved lymph node sampling [18, 19]. Indeed, Valsecchi et al. found that the experience of the pathologist was an independent predictor of the number of lymph nodes sampled [20]. We also found that higher lymph node counts were correlated with younger patient age. In support of this observation, Chou et al. found that the number of lymph nodes sampled was negatively correlated with older age, and they speculated that surgeons may be more likely to perform less extensive resections due to the limited anesthesia time associated with older patients with comorbidities [21]. Tekkis et al. reaffirmed this hypothesis and further hypothesized that lymph nodes in older patients may be subject to involution [22].

There is no well-established “optimal” cutoff ratio for the interpretation of LNR. In fact, the value varies widely from study to study. Ceelen et al., who conducted a systematic review of 16 studies that included a total of 33,984 patients with stage III colorectal cancer, demonstrated that LNR cut-off values range anywhere from 6 to 75% [13]. Almost all of the proposed cut-offs throughout the literature were arbitrary and were mostly classified on Kaplan-Meier plots. The authors recommended an LNR cut-off of 0.10 for any future prospective investigation, which was the median of all documented LNR cut-off values [13]. Nonetheless, Ceelen’s recommendation is as arbitrary as the cut-off values set by other studies, of which only one was calculated on a statistical basis.

Using Ceelen et al.’s proposed cutoff ratio of 0.10, we found that LNR statistically significantly predicted OS in both unadjusted and adjusted analyses. In support, Sabbagh et al. found that a cutoff ratio of 0.10 was optimal according to the results of a ROC analysis. Using this cutoff ratio, they found that LNR significantly predicted OS and disease-free survival [23]. However, our analysis demonstrated that an LNR of 0.16 demonstrated similar prognostic predictive power to Ceelen’s proposed LNR but with higher diagnostic accuracy.

Despite the various cutoff ratios used in the literature, Zhang et al. performed a meta-analysis of 33 studies and concluded that a higher LNR independently predicted survival in patients with colorectal cancer, and LNR should be integrated into a future staging system [24].

We found that LNR separated survival distributions better than N stage. In support, many studies have shown that LNR offers superior prognostication compared with N stage [13, 25]. However, other studies have shown that LNR performs similarly to N stage or is even inferior [26–28]. Interestingly, some authors have proposed hybrid staging systems for node-positive colon cancer that integrate LNR into the TNM staging system, and their findings show that these hybrid staging systems perform better than the TNM staging system in isolation [29–31]. For example, Wang et al. showed that, according to the original TNM staging system, patients with the T3N1M0 subtype of stage IIIB disease survive longer than other patients with stage IIIB disease and are akin to patients with stage IIIA disease. They also showed that T4aN1M0 disease and an LNR > 30% portended a worse survival than T4aN1M0 and an LNR ≤ 30% and actually resembled stage IIIC disease [31]. Therefore, the integration of LNR into the TNM staging system appears to offer superior prognostication.

Our service processed additional specimens in nine cases, and only one case was revised (upstaged from N2a to N2b). Notably, LNR in all nine cases did not cross the cutoff ratio of 0.10. Although the sampling of additional lymph nodes may result in more accurate staging, the impact of restaging on patient management and outcomes is questionable [32, 33]. The effect of additional sampling on LNR is unclear, and should be assessed by further studies.

Our study has several limitations. First, our utilized LNR cutoff value is based on a median split [13]. Median splitting results in balanced groups of patients that are not necessarily prognostically delineated. However, Sabbagh et al. demonstrated that classifying patients into more than two groups obscured the relationship between LNR and survival. In addition, they also showed that a cutoff ratio of 0.10 yielded optimal sensitivity and specificity for the prediction of disease-free survival [23]. Second, our second LNR cut-off of 0.16 may fit only a population with similar characteristics; however, temporal validation of our data showed optimal LNR cut-offs of similar value and diagnostic accuracy. Third, our service sampled additional lymph nodes in only nine cases. Therefore, we could not produce compelling evidence for or against the reliability of LNR with additional sampling; further studies are required to measure the rate of LNR reclassification with additional sampling. Fourth, the moderate size of our sample may have restricted the power of our statistical analysis.

## Conclusions

LNR is a robust predictor of overall survival in patients with stage III colorectal adenocarcinoma. At a cutoff ratio of 0.10 or 0.16, LNR offers better prognostic stratification compared with N stage and is less susceptible to variation introduced by the number of lymph nodes sampled, which is influenced by both clinical characteristics and grossing technique.

## Data Availability

The data used to support the findings of this study are available from the corresponding author upon request.

## Abbreviations

AJCC: American Joint Committee on Cancer
UICC: Union for International Cancer Control
LNR: Lymph node ratio
KHCC: King Hussein Cancer Center
JCI: Joint Commission International
OS: Overall survival
ROC: Receiver operating characteristic
